# Nanotechnology-enabled delivery of luteolin: a comprehensive review on multidisease therapeutic applications

**DOI:** 10.3389/fphar.2026.1792796

**Published:** 2026-02-27

**Authors:** Yutong Li, Hongxuan Chen, Yong Lu, Shenghui Zhong, Xianfu Wu

**Affiliations:** 1 National Institutes for Food and Drug Control, Beijing, China; 2 School of Basic Medical Sciences, Yichun University, Yichun, Jiangxi, China

**Keywords:** disease treatment, luteolin, nanocomposites, potential applications, research progress

## Abstract

Luteolin is a natural flavonoid compound widely found in various plants, known for its antioxidant, anti-inflammatory, cardiovascular protective and anti-tumor activities. However, its low bioavailability due to rapid metabolism and low solubility hinders its clinical application. In recent years, with the development of nanotechnology, luteolin combined with nanomaterials to form nanocomposites has shown promising drug delivery performance and therapeutic effects in numerous diseases. This article reviews the latest research progress on luteolin nanocomposites in disease treatment, exploring their potential applications in cancer, diabetes, cardiovascular diseases, neurological disorders, and other conditions.

## 1 Introduction

Luteolin, an important plant-derived flavonoid compound, has attracted significant attention from researchers due to its extensive pharmacological activities. Studies have shown that luteolin possesses multiple biological activities, including antioxidant, anti-inflammatory, anticancer, and cardiovascular protective effects (Imran et al., 2019; Li B. et al., 2021; Lin et al., 2008). However, its poor water solubility and low bioavailability significantly limit its application in clinical medicine (Hostetler et al., 2017). To overcome this issue, researchers increasingly focused on employing nanotechnology to improve the performance of luteolin.

The application of nanotechnology provides an innovative solution for enhancing the bioavailability of luteolin. By loading luteolin onto various nanocarriers, such as luteolin pure drug nanocrystals, nanocapsules, liposomes, or polymer nanoparticles, its water solubility and stability can be effectively improved (Liu J. et al., 2021; Tawornchat et al., 2021; Xu et al., 2023). These nanocarriers not only prevent the degradation of luteolin but also enhance its absorption and distribution in the body. For instance, Chen et al. prepared luteolin nanoparticles using liquid anti-solvent precipitation and vacuum freeze-dry technology, which effectively increased bioavailability and efficiently inhibited liver microsomal peroxidation in rats (Chen et al., 2021). Furthermore, surface modification techniques of nanocarriers enable more precise targeting of diseased tissues, facilitating targeted therapy and absorption (Shang et al., 2023). In previous studies, Liu et al. designed and prepared quercetin nanocrystals modified with sodium dodecyl sulfate (SDS), which effectively enhanced quercetin’s solubility and the bioavailability of oral formulations (Liu J. et al., 2021). In this study, they compared unmodified nanocrystals with SDS-modified ones, finding that the bioavailability of quercetin in the SDS-modified nanocrystals increased by approximately twofold. This increase may be attributed to SDS’s ability to effectively open tight junctions in the intestine.

Through targeted delivery mechanisms, the accumulation of luteolin in tumor cells and other pathological sites is significantly increased, which not only enhances its therapeutic efficacy but also reduces damage to normal cells, thus lowering side effects. For example, modifying nanocarriers with ligands folic acid specific to tumor cell surfaces allows for precise targeting of tumors (Wang et al., 2021). In this context, the clinical prospects for the application of luteolin are broadening. In addition, studies have indicated that the synergistic effects between luteolin and nanocarriers may enhance its biological activity, leading to stronger effects in the treatment of certain diseases. This review may provides new ideas and directions for future clinical nanodrug development.

## 2 Pharmacological effects of luteolin

Luteolin is a natural flavonoid compound that is widely present in various plants. Recent studies have shown that it exhibits significant pharmacological properties in the prevention and treatment of various diseases (Goyal et al., 2024). The following will discuss in detail the four main effects of luteolin: antioxidant effects, anti-inflammatory effects, and anticancer effects.

### 2.1 Antioxidant effects

The antioxidant effect of luteolin is one of its most well-known pharmacological properties. The production of free radicals is closely related to oxidative stress damage, which is considered an important trigger for various chronic diseases and the aging process (Teleanu et al., 2022; Forman and Zhang, 2021). Research has shown that luteolin effectively removes free radicals generated in the body and enhances the cell’s own antioxidant capacity, thereby reducing the impact of oxidative damage on cells. Specifically, luteolin activates the antioxidant enzyme system, such as superoxide dismutase (SOD) and glutathione peroxidase (GPx), enhancing the cell’s defense mechanism and providing a stronger protective barrier (Gu et al., 2024). This characteristic has attracted significant attention in research related to cardiovascular diseases, diabetes, and neurodegenerative diseases (Chagas et al., 2022).

### 2.2 Anti-inflammatory effects

Luteolin also exhibits significant anti-inflammatory effects. During the process of chronic inflammation, the abnormal release of cytokines plays an important role, with increased levels of pro-inflammatory factors such as tumor necrosis factor-alpha (TNF-α) and interleukin-6 (IL-6) leading to various pathological states in the body. Luteolin can effectively alleviate local and systemic inflammatory responses by inhibiting the secretion of these cytokines (Aziz et al., 2018). Some studies suggest that luteolin may suppress the expression of inflammation-related genes by regulating the nuclear factor kappa B (NF-κB) signaling pathway, thereby reducing the release of inflammatory mediators, which has potential therapeutic value for diseases such as rheumatoid arthritis and inflammatory bowel disease (Caporali et al., 2022; Conti et al., 2021).

### 2.3 Anticancer effects

The anticancer effect of luteolin has attracted widespread attention from research ers in recent years. Multiple experimental results show that luteolin can induce apoptosis in various cancer cells (such as breast cancer, colon cancer, and lung cancer) and inhibit their proliferation (Zhang et al., 2006). The mechanism of this effect may involve the regulation of multiple cell signaling pathways, including the upregulation of pro-apoptotic genes and downregulation of anti-apoptotic gene expression (Lee et al., 2012). Additionally, luteolin may also play an important auxiliary role in anticancer therapy by inhibiting the migration and invasion capabilities of tumor cells, thereby reducing the risk of metastasis (Ashrafizadeh et al., 2020).

### 2.4 Cardiovascular protective effects

Previous studies have shown that luteolin has significant cardiovascular protective effects (Luo et al., 2017). In recent years, increasing evidence supports the mechanism by which luteolin prevents cardiovascular diseases by improving endothelial function and reducing low-density lipoprotein cholesterol (LDL-C) levels (Fikry et al., 2024). First of all, endothelial cells play a crucial role in cardiovascular health. Normal endothelial function helps maintain the balance of vasodilation and vasoconstriction, regulates blood pressure, and prevents the occurrence of atherosclerosis. Studies have shown that luteolin can enhance the synthesis and release of nitric oxide (NO) in endothelial cells, promoting vasodilation, thereby improving microcirculation and reducing the risk of cardiovascular diseases. In addition, luteolin can also inhibit the inflammatory response of endothelial cells, further protecting the integrity of the vascular endothelium and lowering the incidence of cardiovascular diseases (Wang et al., 2023). At the same time, high levels of LDL cholesterol are considered an important risk factor for cardiovascular diseases. Luteolin effectively lowers LDL cholesterol levels by influencing cholesterol metabolism, particularly by regulating the liver’s absorption and excretion of cholesterol (Orrego et al., 2009). Additionally, luteolin may also reduce oxidative stress through its antioxidant properties, which is particularly important for preventing arterial damage caused by high cholesterol. Therefore, the application of luteolin is seen as a promising strategy for improving cholesterol levels and reducing the risk of cardiovascular diseases.

However, the bioavailability of luteolin is relatively low, which limits its effectiveness in clinical applications. To address this issue, the application of nanocomposites has emerged. Nanotechnology can enhance the accumulation concentration of drugs in specific tissues, thereby improving their bioavailability and efficacy. By encapsulating luteolin in nanocarriers, its concentration in cardiac tissue can be significantly increased (Pechanova et al., 2020). This not only enhances the cardioprotective effects of luteolin but also provides new ideas and directions for the treatment of cardiovascular diseases.

## 3 Application of luteolin nanocomposites in disease treatment

### 3.1 Luteolin nanocomposites for cancer treatment

Luteolin-based nanocomposites have shown remarkable efficacy in cancer treatment. Previous studies have demonstrated that luteolin, as an active ingredient, effectively inhibits tumor cell growth by inducing apoptosis and suppressing angiogenesis (Lin et al., 2008). The use of nanocarrier technology significantly enhances the targeted delivery of luteolin, simultaneously reducing damage to normal tissues. The nano-formulations also exhibit multifaceted synergistic effects in cancer therapy research, primarily through mechanisms such as enhancing chemotherapeutic efficacy, remodeling the tumor immune microenvironment, reversing drug resistance, and inhibiting tumor angiogenesis (Wang et al., 2021). Research on these nanocomposites offers new perspectives and possibilities for developing safer and more effective cancer treatment strategies.

**FIGURE 1:**
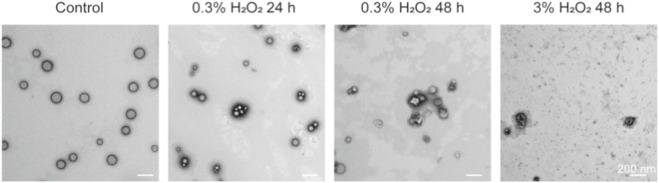
Electron micrographs of PPS-NPs degradation after hydrogen peroxide treatment. Reproduced from ref (Fu et al., 2023). Copyright 2023 Taylor and Francis.

Fu et al. prepared ROS-responsive resveratrol nano-composites using the double emulsion solvent volatilization method, LUT@PPS-PEG nano-composites, with a particle size of 169.77 ± 7.33 nm (Fu et al., 2023). The drug delivery material in this system is the common reactive oxygen species-responsive material Poly (propylene sulfide)-poly (ethylene glycol) (PPS-PEG). *In vitro* release experiments showed that LUT@PPS-PEG could indeed achieve ROS-responsive release of resveratrol (Figure 1). In further pharmacological experiments, LUT@PPS-PEG nano-composites were effectively taken up by SK-MEL-28 cells and significantly inhibited their proliferation and migration. Animal experiments also demonstrated good anti-melanoma effects (Fu et al., 2023).

**FIGURE 2:**
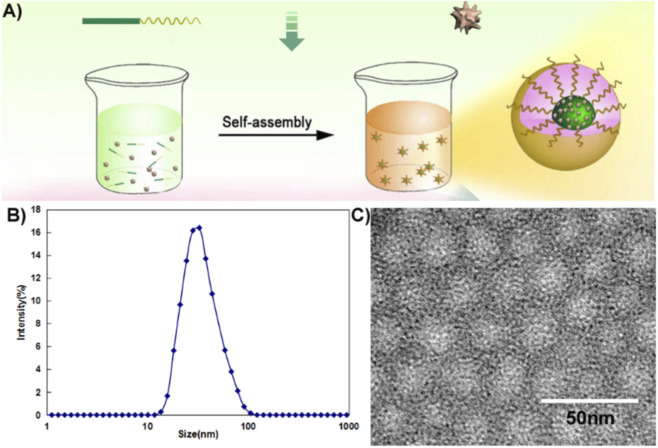
**(A)** Preparation of Luteolin/MPEG-PCL micelles. Luteolin/MPEG-PCL micelles were prepared by self-assembly methods. First, luteolin and MPEG-PCL were co-dissolved in acetone. Then the mixture was added into water, and in this process luteolin and MPEG-PCL assembled into micelles with luteolin and PCL inside and PEG outside. **(B)** The size of Luteolin/MPEG-PCL micelles. **(C)** Transmission electron microscopy (TEM) image of Luteolin/MPEGPCL micelles. Reproduced from ref (Wu et al., 2019). Copyright 2019 Taylor and Francis.

Wu et al. designed and prepared folacin-modified luteolin-loaded nanocomposites, Luteolin/Fa-PEG-PCL, for glioma therapy (Wu et al., 2019). This drug delivery system uses folic acid modified poly (ethylene glycol)-poly (ε-caprolactone) (Fa-PEG-PCL) nano-micelles to encapsulate luteolin (Figure 2). The modification with folic acid is able to increase tumor cell uptake of this nanomedicine, thereby enhancing targeting specificity and improving efficacy. Subsequent *in vitro* and *in vivo* experiments demonstrated that Luteolin/Fa-PEG-PCL significantly inhibited the proliferation of GL261 cells and induced apoptosis. In animal experiments, it was found that Luteolin/Fa-PEG-PCL accumulated at the glioma site and significantly inhibited glioma growth. In another study focusing on glioma, Zheng et al. designed and prepared luteolin-loaded MPEG-PCL nanomicelles. These nanomicelles significantly improved the solubility and bioavailability of luteolin, and achieved sustained release of the compound. They further validated the anti-tumor effects of this formulation at the cellular and animal levels, showing that compared to free luteolin, luteolin-loaded MPEG-PCL nanomicelles could more effectively induce apoptosis in C6 and U87 cells and inhibit neovascularization in tumor tissues. Additionally, further cell experiments indicated that these nanomicelles induced apoptosis in tumor cells through downregulation of Pro-caspase9 and Bcl-2 and upregulation of cleaved-caspase9 and Bax via the mitochondrial pathway. Animal distribution experiments demonstrated that luteolin-loaded MPEG-PCL nanomicelles could accumulate in tumor tissues, achieving tumor targeting (Wu et al., 2019).

In a therapeutic study targeting breast cancer, Wang et al. designed and prepared f olic acid-modified ROS-responsive nanoparticles for targeted delivery of luteolin, based on the characteristics of high levels of ROS in the tumor microenvironment and high expression of folate receptors on tumor cells (Wang et al., 2021). They first synthesized ROS-responsive materials (Oxi-aCD) and then obtained core-shell nanoparticles using a nanoprecipitation/self-assembly method. These were further modified with DSPE-PEG-FA to obtain the final nanocomposite Lut/FA-Oxi-aCD. The nanocomposite had a relatively uniform particle size of 196.7 nm. In drug release experiments, results showed that this system could achieve ROS-responsive release of luteolin. Subsequent cell experiments demonstrated that Lut/FA-Oxi-aCD was efficiently internalized by 4T1 cells and significantly inhibited their proliferation. Final animal experiments also proved that Lut/FA-Oxi-aCD possessed long circulation and tumor-targeting properties, with significant tumor inhibition effects (Wang et al., 2021). As shown in Table 1, we have summarized the recent research applications and associated functional properties of luteolin nanoformulations in cancer therapy.

**TABLE 1:** Common nanoformulations of luteolin for cancer treatment.

Carriers	Tumor category	Properties	Ref
PLGA/Liposome nanoparticles (NPs) modified with PD-L1 antibody	Liver cancer	Significantly reduce the expression of Bcl-2 and increase the level of LDH in cells; Enhance the uptake of luteolin	Cao and Wang (2021)
Ethosomes	Hepatocellular carcinoma	Improve hepatic adenomas and reduce neoplastic hepatic lesions	Elsayed et al. (2021)
Luteolin functionalized zinc oxide nanoparticles	Lung cancer	Autophagy activation and EMT inhibition	Gao et al. (2024)
*In situ* gel containing luteolin micelles	Colorectal cancer peritoneal metastasis	Extend drug release; induce tumor cell apoptosis; thermosensitive	Hu et al. (2020)
Effervescent-based nanocarrier	Pancreatic cancer	Disassembles at intestinal pH and is absorbed through the intestinal lymphatic system to further site-specifically invade the pancreatic cancer cells; Activate caspase-3 activity and downregulate VEGF-A, FAK, TNF-α, and Ki-67	Karole et al. (2024)
Folic acid-modified ROS-responsive nanoparticles (Lut/FA-Oxi-aCD NPs)	Breast cancer	Specially accumulate at tumor sites and inhibit tumor growth	Wang et al. (2021)
Folic acid modifiedpoly (ethylene glycol)-poly (e-caprolactone) (Fa-PEG-PCL) nano-micelles	Glioma	Induce a significant cell growth inhibition and apoptosis of GL261 cells	Wu et al. (2019)
MPEG-PCL micelles	Glioma	Release luteolin in a sustained manner *in vitro*; induce glioma cell apoptosis via the mitochondrial pathway	Zheng et al. (2017)
Poly (propylene sulfide)-poly (ethylene glycol) (PPS-PEG) coating luteolin nanoparticles	Melanoma	High drug loading; Inhibited tumor cell proliferation, migration and invasion	Fu et al. (2023)

### 3.2 Luteolin nanocomposites for the treatment of diabetes and its complications

Diabetes has become an increasingly severe health issue worldwide, with its incidence continuously rising (Glovaci et al., 2019). In addition to classic metabolic disorders, there is growing evidence indicating a significant association between diabetes and neurological diseases (Campayo et al., 2011). In particular, the common occurrence of brain inflammation in diabetic patients has drawn widespread attention from researchers. This neuroinflammation may not only affect cognitive function but also exacerbate patients’ conditions by promoting the development of neurodegenerative diseases (Chong et al., 2024; Kopp et al., 2022). Regarding this situation, Moustafa et al. developed a ZnO nanoformulation loaded with luteolin that can attenuate neuroinflammation associated with diabetes, and used this formulation to treat a diabetic rat model. The results show that luteolin/ZnO nanoparticles can reduce lipid peroxidation, increase antioxidant enzyme activity, and decrease inflammation under oxidative stress. Therefore, they can lower hyperglycemia, enhance insulin levels, and improve insulin resistance. In addition, luteolin/ZnO nanoparticles upregulate miR-124, decrease C/EBPA mRNA, increase Bcl-2, and inhibit apoptosis. The results suggest that diabetes increases blood-brain barrier permeability through the downregulation of tight junction proteins, a phenomenon that is restored following treatment with luteolin/ZnO nanoparticles. Luteolin/ZnO nanoparticles target C/EBPA to regulate miR-124 and microglial cell polarization, and alleviate inflammatory damage by modulating oxidative stress-sensitive signaling pathways. Luteolin/ZnO nanoparticles present a novel therapeutic target for protecting the blood-brain barrier and preventing diabetic neurovascular complications (Moustafa et al., 2023).

Parra et al. synthesized selenium nanoparticles co-loaded with diosmin and luteolin (LU/DIO-SeNPs), with an average particle size of 47.84 nm (Gutiérrez et al., 2022). They subsequently applied this nanomedicine for the treatment of streptozotocin-induced diabetic mice. Compared to STZ-induced untreated diabetic mice, significant changes were observed in body weight, food intake, and water consumption. Additionally, LU/DIO-SeNPs exhibited strong antioxidant activity, as evidenced by the detection of catalase (CAT), superoxide dismutase (SOD), and glutathione peroxidase (GPx) in the liver and kidneys. These nanoparticles also demonstrated a preventive effect against liver damage, assessed through the activity of aspartate aminotransferase (AST), alanine aminotransferase (ALT), and alkaline phosphatase (ALP). The nanospheres showed remarkable antidiabetic activity, with a synergistic effect between selenium and flavonoids. This study presents a novel type of SeNPs nanosphere prepared using an efficient strategy, designed to incorporate rutin and quercetin to enhance the efficacy against type 2 diabetes (Gutiérrez et al., 2022).

Diabetic ureteral injury is a relatively rare but clinically significant complication that is closely associated with the long-term hyperglycemic state in diabetic patients (Yuan et al., 2023). This condition is usually caused by microvascular and neuropathic changes due to diabetes, which significantly affect the structure and function of the urinary system. The pathogenesis of diabetic ureteral injury mainly involves vascular endothelial damage and nervous system impairment induced by hyperglycemia. Chronic hyperglycemia can lead to thickening of the microvascular basement membrane and dysfunction of vascular endothelial cells, resulting in insufficient local blood supply. Additionally, diabetes can cause autonomic neuropathy, affecting the peristaltic function of the ureter, leading to urine retention or reflux, thereby increasing the risk of urinary tract infections and chronic kidney damage (Agashe and Petak, 2018). Moreover, oxidative stress under hyperglycemic conditions can directly damage the epithelial cells of the ureter (Jolivalt et al., 2016). Jing et al. prepared selenium nanoparticles loaded with luteolin (Luteolin-SeNPs) and used them for the treatment study of diabetic ureteral injury. Cytotoxicity assays on HEK 293 and NIH-3T3 showed >90% cell viability, demonstrating excellent biocompatibility of the formulation. Further research has shown that Luteolin-SeNPs can inhibit the NLRP3(NOD-like receptor family pyrin domain containing 3) inflammasome through the Nrf2/ARE pathway, which is beneficial for the treatment of diabetic ureteral injury. The developed luteolin-SeNPs may become an effective formulation for the treatment of diabetic ureteral injury (Jing et al., 2024).

### 3.3 Luteolin nanocomposites for the treatment of neurological disorders

The pathogenesis of neurodegenerative diseases is complex, and there is currently no effective cure (Temple, 2023; Chi et al., 2018). Therefore, finding novel and effective therapeutic strategies is of paramount importance. Luteolin, with its unique biochemical properties and multiple biological activities, has become a research hotspot in this field. Luteolin, a flavonoid compound, exerts neuroprotective effects through various mechanisms (He et al., 2023). On the one hand, it can reduce intracellular reactive oxygen species (ROS) through its antioxidant activity, thereby mitigating oxidative stress-induced damage to neural cells (Jayawickreme et al., 2024). On the other hand, luteolin can inhibit neuroinflammatory responses, regulate intracellular signaling pathways, and promote the release of neurotrophic factors, thus preserving neuronal function (Kempuraj et al., 2021). Furthermore, luteolin has been found to inhibit the aggregation of β-amyloid proteins, an action of significant importance in the prevention and treatment of Alzheimer’s disease (Younis et al., 2024).

AD is often characterized by the advanced deterioration of cognition and memory, and it accounts more than 60% of most dementia cases (Scheltens et al., 2021). The presence of oxidative stress markers is one of the earliest changes that occur in the brains of individuals with AD (Bai et al., 2022; Chen and Zhong, 2014). As mentioned earlier, luteolin has various activities such as antioxidant properties, and thus it is gaining more attention in the treatment of AD. However, due to the presence of the blood-brain barrier, enhancing the concentration of luteolin within the brain is a major challenge for its clinical application (Passeri et al., 2022). To address this, Abbas et al. designed and prepared novel luteolin-loaded chitosan-decorated nanoparticles that can target the brain. Behavioral studies indicate significant improvement in the acquisition of both short-term and long-term spatial memory in mice. Furthermore, histological assessments reveal increased neuronal survival rates and a reduction in the number of amyloid plaques. Biochemical results demonstrate enhanced antioxidant effects and reduced levels of pro-inflammatory mediators. Additionally, compared with the model control group, the reduction in Aβ aggregation and hyperphosphorylated tau protein levels reached 50%, further confirming the ability of luteolin-loaded chitosan nanoparticles (LUT-CHS) to alleviate pathological changes associated with AD (Abbas et al., 2022).

In another study on AD treatment, Elsheikh et al. developed via Intranasal Luteolin-Loaded Nanobilosomes. The Nanobilosomes were prepared using the thin-film hydration technique, with a particle size of 153.2 ± 0.98 nm, and a loading efficiency of Luteolin reaching 70.4% ± 0.77%. The *in vivo* therapeutic effect experiment was conducted on AD mice models, which was established via intracerebroventricular injection of 3 mg/kg of streptozotocin. Behavioral, biochemical, histological, and immunohistochemical experiments were performed after 21 days of intranasal inhalation of luteolin bilosomes or luteolin suspensions (50 mg/kg). The luteolin bilosomes improved short-term and long-term spatial memory and also showed antioxidant properties, reducing the levels of pro-inflammatory mediators. They also inhibited the aggregation of amyloid βand the overphosphorylation of Tau proteins in the hippocampus. Compared to luteolin suspension, luteolin bilosomes represent an effective, safe, non-invasive approach with superior cognitive function (Elsheikh et al., 2022).

### 3.4 Luteolin nanocomposites for the treatment of other disease

Luteolin nanocomposites, as a novel drug carrier, have demonstrated broad application prospects in disease treatment. In addition to their performance in the aforementioned diseases, they have also shown good effects in the treatment of diabetes, liver diseases, and immune system disorders.

#### 3.4.1 Luteolin nanocomposites for the treatment of non-alcoholic fatty liver disease

Non-alcoholic fatty liver disease (NAFLD) is a global health issue with high prevalence and incidence, closely associated with obesity, insulin resistance, type 2 diabetes (T2DM), and dyslipidemia (Pouwels et al., 2022; Maurice and Manousou, 2018). Given the strong link between NAFLD and T2DM, the most common treatment approach for NAFLD involves the use of anti-diabetic drugs (Pouwels et al., 2022; Huang et al., 2020). However, oral hypoglycemic agents come with certain side effects. Therefore, a diet rich in natural plant nutrients is also effective for diabetes management and for patients with NAFLD, as these plant nutrients possess various pharmacological activities (Akhlaghi, 2016; Wang et al., 2020; Yang et al., 2022). As a result, the naturally abundant compound luteolin has emerged as a potential treatment for NAFLD. By ingesting luteolin, it is possible to reduce hepatic fat accumulation, improve insulin sensitivity, and exert beneficial effects on inflammation and oxidative stress, making it a promising new strategy for the treatment of NAFLD (Liu et al., 2014; Yin et al., 2017; Liu X. et al., 2021). In response to this situation, Ahmed et al. developed luteolin loaded on zinc oxide nanoparticles ameliorates non-alcoholic fatty liver disease associated with insulin resistance in diabetic. They prepared Luteolin/ZnO nanocomposites with a particle size of 174.7 nm using the One pot method, and subsequently used them for the treatment of a diabetic rat model with NAFLD. The results showed that Luteolin/ZnO nanoparticles effectively reduced hyperglycemia and hyperinsulinemia and improved insulin resistance. Additionally, Lut/ZnO nanoparticles improved liver function, enhanced the antioxidant system, and reduced oxidative stress markers. Furthermore, by inhibiting lipogenesis and gluconeogenesis, the lipid load in the liver and the levels of triglycerides and total cholesterol in circulation were decreased. Moreover, Lut/ZnO nanoparticles activated the PI3K/AKT signaling pathway, leading to the inactivation of FoxO1, which enhanced insulin sensitivity in hepatocytes (Ahmed et al., 2022).

#### 3.4.2 Luteolin nanocomposites for the treatment of ulcerative colitis

Ulcerative colitis (UC) is a complex chronic disease and a subtype of inflammatory bowel disease (IBD), primarily affecting the intestinal mucosa and submucosal layers of the rectum and colon (Adams and Bornemann, 2013; Du and Ha, 2020). This disease not only presents significant clinical symptoms such as abdominal pain and bloody diarrhea but, due to its chronic nature, can lead to long-term nutritional absorption issues, weight loss, and a decline in quality of life. In recent years, the incidence of UC has rapidly increased worldwide, especially in emerging industrialized countries, drawing widespread attention from both the academic and medical communities. Although researchers have yet to fully clarify the pathogenesis of UC, an increasing body of research suggests that the interaction between environmental factors and the gut microbiome may play a crucial role in the development of the disease (Ordás et al., 2012; Eisenstein, 2018; Pabla and Schwartz, 2020). In individuals with genetic susceptibility, these factors can trigger abnormal immune-inflammatory responses, which disrupt the intestinal barrier function, leading to mucosal damage and inflammation. Additionally, the imbalance in the gut microbiota composition is considered a key biological marker in the disease process of UC, and may even serve as a potential therapeutic target.

Existing reports indicate that the gastrointestinal structure and function of IBD patients undergo continuous and irreversible damage throughout the course of the disease, significantly increasing the risk of colon cancer (Yadav et al., 2022). Therefore, early diagnosis and appropriate intervention are critical for improving patient prognosis. Currently, despite the availability of various treatments, including 5-aminosalicylic acid drugs, corticosteroids, immunomodulators, and biologics, the effectiveness of existing treatments is only about 40%, according to statistical data (Turner et al., 2018; Segal et al., 2021; Keshteli et al., 2019). This highlights the urgent need for the development of new therapies. In studies on UC, ROS are considered one of the key factors influencing the pathological process (Chen et al., 2020; Wan et al., 2022; Han et al., 2023). ROS are naturally produced molecules during cellular metabolism, but their excessive accumulation in the body can lead to a range of biological effects, including damage to cell membranes, protein oxidation, and DNA breakage. Particularly in the intestinal tract, excessive ROS generation can not only trigger localized inflammatory responses but may also activate the immune system, leading to systemic immune abnormalities that exacerbate the symptoms and severity of UC (Wan et al., 2022). Therefore, regulating ROS has become a novel strategy for preventing and treating UC.

Existing research indicates that inhibiting the generation of ROS can effectively reduce inflammatory responses and improve intestinal function in UC patients (Ma et al., 2018). In recent years, many antioxidants have garnered attention for their ability to lower ROS levels in the body. These antioxidants can act by directly scavenging free radicals or by modulating the activity of antioxidant enzymes and altering related signaling pathways to suppress ROS production (Liu et al., 2020). For instance, luteolin, a natural plant-derived antioxidant, has shown positive effects on intestinal health in numerous studies. Luteolin not only exhibits potent antioxidant capabilities but also reduces tissue ROS levels by regulating the expression of inflammatory factors, thereby alleviating UC symptoms (Li B. et al., 2021; Li B. L. et al., 2021).

Based on the above situation, Tan et al. designed and developed a reactive oxygen species-responsive nanoscale drug delivery system using phyllanthin. The nano-drug delivery system is prepared using D-α-tocopherol polyethylene glycol succinate-b-poly (β-thioester) copolymer (TPGS-PBTE), designed to target the delivery of luteolin for the cleavage of ROS. Due to the sulfur ether bonds in the polymer backbone, TPGS-PBTE nanoparticles exhibit responsive size changes and drug release in the presence of ROS, which aids in the clearance of ROS in inflamed colons and selective accumulation of Luteolin. In the acute colitis mouse model induced by sodium carboxymethyl cellulose, Luteolin@TPGS-PBTE nanoparticles alleviated weight loss, shortened colon length, and colon tissue damage caused by ROS and pro-inflammatory cytokines (such as IL-17A, IL-6, interferon-γ, and TNF-α), while upregulating glutathione and anti-inflammatory factors (such as IL-10, IL-4). More importantly, Luteolin@TPGS-PBTE nanoparticles modulate the inflammatory microenvironment by regulating the Th1/Th2 and Th17/Treg cell balance (i.e., increasing the number of Treg and Th2 cells and decreasing the number of Th1 and Th17 cells), thus alleviating inflammation and accelerating mucosal healing in the intestine. Furthermore, the Luteolin@TPGS-PBTE nanoparticle formulation reduces the effective dose of luteolin and demonstrates excellent biocompatibility in the mouse model, highlighting its potential as an oral formulation for targeted treatment of ulcerative colitis (Tan et al., 2022).

#### 3.4.3 Luteolin nanocomposites for the treatment of hyperuricemia

Hyperuricemia often has no obvious symptoms, but when uric acid levels rise significantly, it can trigger gout, a disease characterized by acute arthritis (Yanai et al., 2021). Gout usually occurs with severe joint pain, commonly affecting the big toe, knee joints, and wrist joints. Long-term hyperuricemia not only increases the frequency of gout attacks but may also lead to complications such as kidney stones and impaired kidney function (Zhang et al., 2019; Petreski et al., 2020). Studies have shown that luteolin may exert some protective effects on hyperuricemia and its related complications by inhibiting uric acid production and improving kidney function (Adachi et al., 2021; Lin et al., 2018; Tang et al., 2023). Luo et al. developed an oral formulation of luteolin nanocrystals stabilized with Hydroxyethyl Starch (LUT-HES Nanocrystals) for the treatment of hyperuricemia. The particle size of LUT-HES Nanocrystals is 191.1 ± 16.8 nm, exhibiting extremely high encapsulation efficiency and drug loading capacity, which are 98.52% ± 1.01% and 49.26% ± 0.50%, respectively. In treatment studies on hyperuricemic mice, it was shown that compared to the coarse drug, LUT-HES Nanocrystals significantly enhance the oral bioavailability of the drug. Pharmacodynamic studies demonstrated that LUT-HES Nanocrystals remarkably reduced serum uric acid levels by 69.93% and improved renal damage in hyperuricemic mice (Luo et al., 2024).

## 4 Perspectives and summary

Numerous existing studies have demonstrated that luteolin has a wide range of pharmacological effects and can be used as a therapeutic or adjuvant drug for many diseases. The nanocomposites of luteolin, as an emerging drug delivery system, demonstrate good potential for clinical applications. The nano-delivery system enhances the solubility and bioavailability of luteolin by encapsulating it in a nano-carrier (Liu J. et al., 2021). These nano-carriers can be biocompatible materials, such as polymers, lipids, and inorganic nanoparticles, which not only protect luteolin from degradation by enzymes in the body but also enable targeted drug delivery, which improves efficacy and reduces side effects. Numerous researches indicate that luteolin nanocomposites exhibit significant efficacy in both *in vitro* and *in vivo* experiments (Espíndola, 2023; Kar et al., 2024). For instance, in cancer therapy, these nanocomposites can effectively inhibit the proliferation of tumor cells and induce apoptosis while having minimal impact on normal cells. Although luteolin nanocomposites are very attractive in disease treatment, the potential *in vivo* safety issues of some nanomaterials still deserve attention (Najahi-Missaoui et al., 2020).

In summary, luteolin nanocomposites represent a promising drug delivery system that showcases strong potential for clinical applications. As research continues to advance, the prospects for clinical applications of these nano-complexes will broaden, offering new therapeutic options for various refractory diseases. Future studies should further explore the actual effects and safety of these nanocomposites in clinical treatments to facilitate their translation into clinical practice. Additionally, it is essential to investigate their metabolic pathways and potential toxicity with long-term use to ensure their safety in humans.
